# Predicting induction chemotherapy response based on tumor-stroma ratio and pretreatment synthetic MRI in nasopharyngeal carcinoma

**DOI:** 10.1186/s41747-026-00683-5

**Published:** 2026-02-24

**Authors:** Huanhuan Ren, Xin Zhang, Qian Xu, Daihong Liu, Xinyu Chen, Yao Huang, Hua Lan, Lifeng Li, Yuanyuan Li, Haiping Huang, Jiangdong Sui, Junhao Huang, Xinying Ren, Yao Huang, Yong Tan, Hong Yu, Xiaolei Shu, Yuwei Wang, Huan Zhang, Dan Li, Lisha Nie, Jiuquan Zhang

**Affiliations:** 1 Department of Radiology, Chongqing University Cancer Hospital, Chongqing, China; 2 Radiation Oncology Center, Chongqing University Cancer Hospital, Chongqing, China; 3 School of Medicine, Chongqing University, Chongqing, China; 4 Department of Pathology, Chongqing University Cancer Hospital, Chongqing, China; 5 The School of Medical Imaging, Changsha Medical University, Changsha, China; 6 Hunan Provincial University Key Laboratory of the Fundamental and Clinical Research on Neurodegenerative Diseases, Changsha, China; 7 GE HealthCare MR Research, Beijing, China

**Keywords:** Induction chemotherapy, Magnetic resonance imaging, Nasopharyngeal carcinoma, Nomogram, Patient selection

## Abstract

**Objective:**

There is no satisfactory model for predicting the therapeutic response to chemotherapy of nasopharyngeal carcinoma (NPC). We developed a nomogram using tumor-stroma ratio (TSR) and histogram features from pretreatment synthetic magnetic resonance MRI (SyMRI) to assess induction chemotherapy (IC) response in NPC.

**Materials and methods:**

Data from 185 NPC patients were retrospectively collected from July 2022 to November 2023 (training cohort), and 82 NPC patients were prospectively enrolled from December 2023 to July 2024 (test cohort). A nomogram was developed to predict IC response using logistic regression based on clinicopathological and imaging features from SyMRI T1-, T2-, and proton density (PD)-weighted images, and apparent diffusion coefficient (ADC) maps. The nomogram was validated in the test cohort.

**Results:**

Among the 267 patients (187 males, 80 females), with a mean age of 52.2 years (ranging 43.5–58.7), 181 were responders. Histogram features from ADC and T2-map did not differentiate non-responders (all *p* ≥ 0.220). A clinicopathological model based on TSR and a SyMRI model using T1map_mean and PDmap_Kurtosis were developed. In the test cohort, The nomogram, combining TSR, T1map_mean, and PDmap_Kurtosis, achieved an area under the curve (AUC) of 0.836 (95% CI: 0.690–0.914), outperforming the clinicopathological model (AUC of 0.711, 95% CI: 0.577–0.809, *p* = 0.015) and SyMRI model (AUC of 0.774, 95% CI: 0.623–0.822, *p* = 0.003).

**Conclusion:**

The nomogram combining TSR and histogram parameters from pretreatment SyMRI showed a good performance in predicting IC response for NPC, superior to those of clinicopathological and SyMRI models.

**Relevance statement:**

A nomogram based on pretreatment synthetic MRI and clinicopathological features can help in selecting patients as candidates for IC.

**Key Points:**

- NPC patients with high TSR demonstrated sensitivity to IC.
- The nomogram, integrating TSR and synthetic MRI parameters, achieved a significantly high predictive performance.
- The nomogram may be a reliable tool for predicting the response to IC.

**Graphical Abstract:**

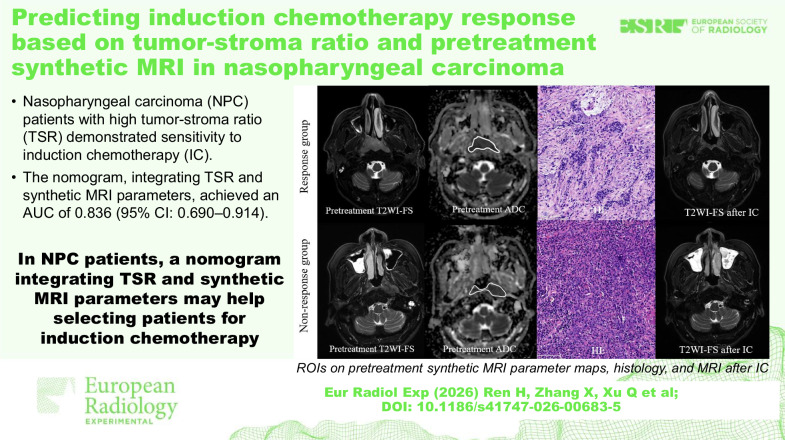

## Background

Concurrent chemoradiation is the standard treatment for locally advanced nasopharyngeal carcinoma (NPC), as recommended by established treatment guidelines [1, 2]. Meta-analyses and phase III clinical trials have demonstrated that induction chemotherapy (IC) can improve survival outcomes in patients with locoregionally advanced NPC compared with those receiving concurrent chemoradiotherapy alone [3]. The response to IC is also a crucial prognostic indicator for NPC [4, 5]. However, only 68–95% of patients achieve an optimal response to IC [6]. Therefore, accurately identifying non-responders before the initiation of treatment could allow for personalized therapy selection and reduce chemotherapy-related toxicity.

The inherent lag in tumor response assessment using Response Evaluation Criteria in Solid Tumors (RECIST) reduces its value in clinical practice. Several predictive models based on MRI have been developed to assess chemotherapy efficacy using various techniques, including intravoxel incoherent motion (IVIM) [7], amide proton transfer [8], and arterial spin labeling [9]. While these studies have demonstrated good predictive performance of the predictive models, their clinical generalizability remains constrained by relatively limited sample sizes (≤ 70 participants).

Currently, tumor-stroma ratio (TSR) has emerged as a reliable stroma-related marker for treatment outcomes and prognosis across various tumor types [10, 11], including head and neck squamous cell carcinoma [12]. However, its significance in predicting IC response in NPC remains limited.

Synthetic magnetic resonance imaging (SyMRI) is a novel, noninvasive quantitative technology that simultaneously generates longitudinal (T1) and transverse (T2) relaxation times, as well as proton density (PD) measurements in a single scan within a few minutes [13], and does not require contrast agent administration. T1, T2, and PD reflect the intrinsic properties of tissue, independent of MRI device specifications or protocols. Studies have demonstrated the value of SyMRI in breast and rectal cancers [14–16], suggesting that T1, T2, and PD provide valuable pathological information for assessing chemotherapy response and prognosis in oncology. Additionally, while the apparent diffusion coefficient (ADC) is crucial in routine MRI, its predictive value in NPC IC response has been reported to be inconclusive [17, 18]: Zheng et al found responders have higher baseline ADC values [17], while others found no significant differences between response and non-response groups [18].

In this study, therefore, we aimed to explore the potential use of histogram parameters from T1, T2, PD, and ADC for the pretreatment IC response prediction in NPC and developed a nomogram to validate the model performance.

## Materials and methods

This study, comprising both retrospective and prospective components, was approved by the ethics review board of our institution. Written informed consent was waived for the retrospective cohort and obtained from all prospective participants before their enrollment in the study. This study adhered to the ethical principles outlined in the Declaration of Helsinki.

Technical support was provided by an author (L.N.), an MRI research scientist at GE Healthcare, adhering to institutional collaboration protocols. This non-remunerative assistance involved no personal interest in study outcomes, maintaining research integrity through complete independence from commercial influence.

### Study sample

**Fig. 1:**
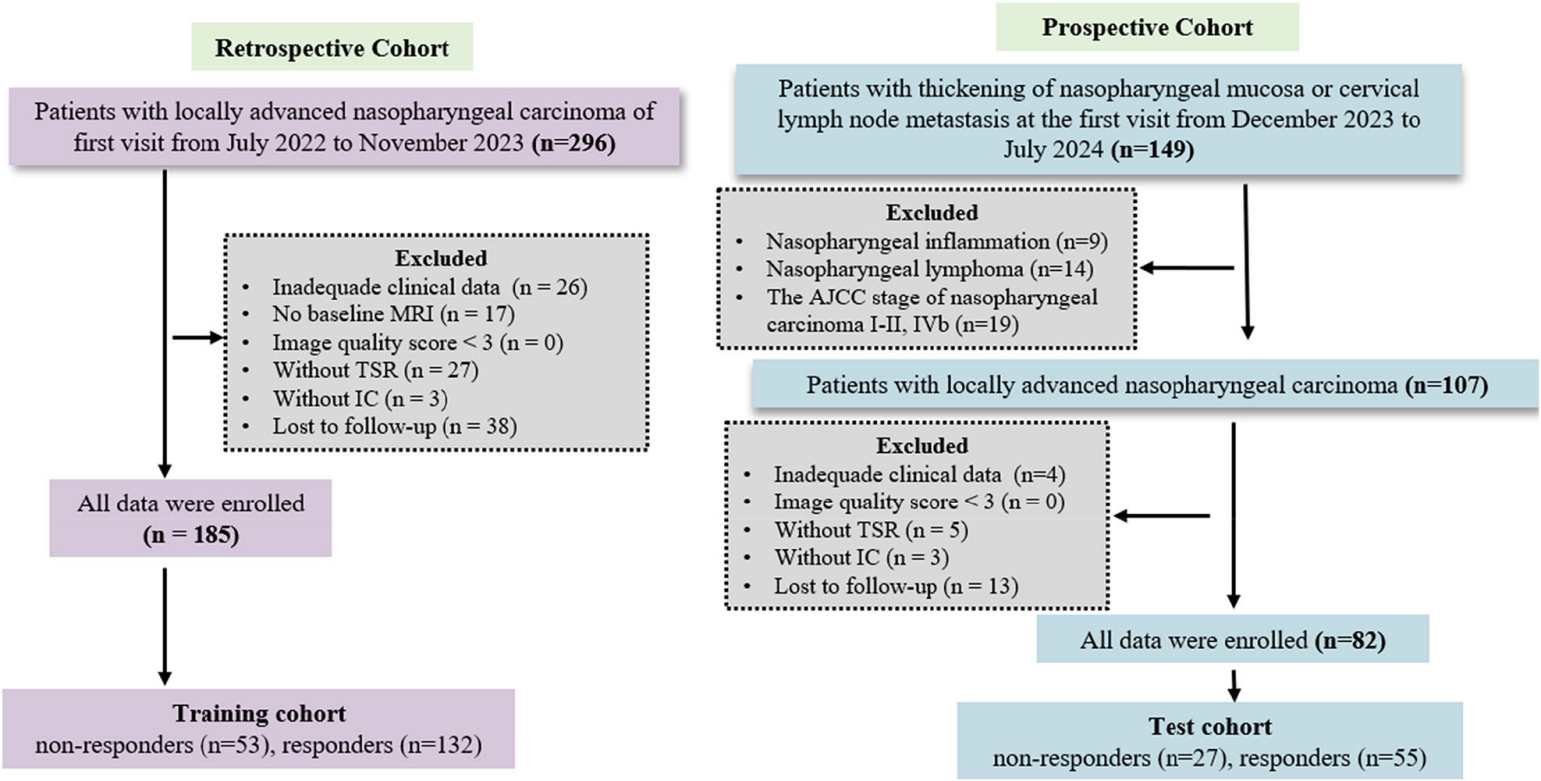
Flowchart of patient selection. IC, Induction chemotherapy; TSR, Tumor-stroma ratio

A retrospective study was conducted on consecutive patients with biopsy-confirmed NPC at our institution between July 2022 and November 2023. These patients constituted the training cohort for this study. The inclusion criteria were: (1); age from 18 to 75 years; (2) pathologically confirmed NPC stage III or IV-A NPC classified according to the 8th American Joint Committee on Cancer staging system [19]; (3) pretreatment nasopharynx and neck MRI, including diffusion-weighted imaging (DWI) and SyMRI; and (4) follow-up MRI images; (5) complete clinical data (including patient sex, age, pretreatment neutrophil, lymphocyte, and platelet count, and Epstein–Barr virus deoxyribonucleic acid copies/mL). Exclusion criteria comprised: (1) non-receipt of IC; (2) inadequate baseline MRI quality (image quality scores < 3 [20]); (3) absence of follow-up MRI; and (4) unavailable TSR results. Additionally, prospective consecutive enrollment (from December 2023 to July 2024) established the test cohort (Fig. 1).

All MRI examinations were performed using a 3-T scanner (Premier, GE Healthcare) with a 32-channel head and neck phased array coil. Scanning parameters are outlined in the Supplementary Table S1.

### TSR assessment

TSR was independently assessed by two pathologists (X.C. and H.H., with 9 and 16 years of experience, respectively) on conventional hematoxylin and eosin-stained slides of NPC puncture samples following the established guidelines [21]. The method for determining high and low TSR groups was provided in the Supplementary Material. In cases of disagreement, consensus was reached through discussion. Pathologists maintained blinding to clinical/imaging data during pathological stratification of participants into stromal-rich (TSR < 50%) and stromal-poor (TSR ≥ 50%) categories [21]. This validated 50% threshold demonstrates established prognostic utility across malignancies, including head and neck squamous cell carcinoma [12] and NPC [21].

### Image processing

ADC maps were automatically generated from DWI data on the scanner console. The raw MAGIC data were then analyzed using SyMRI 8.0 software to produce quantitative T1, T2, and PD maps. Radiologist 1 (with 9 years of experience in head and neck radiology) manually delineated the volumes of interest (VOIs) for the primary tumors on ADC and synthetic T2-weighted images, referring to T2-weighted imaging with fat saturation and contrast-enhanced T1-weighted using ITK-SNAP software (version 4.0, http://www.itksnap.org/). Artifacts and necrotic regions were excluded from their analyses. The VOI delineation was then reviewed by senior radiologist 2 (with 13 years of experience), and in ambiguous cases, consensus was achieved through discussion. Both radiologists were blinded to the clinicopathological data.

The VOIs identified on the synthetic T2-weighted images were copied to the T1, T2, and PD maps. Histogram features including the 10th and 90th percentiles, energy, entropy, interquartile range, kurtosis, maximum, minimum, mean, median, mean absolute deviation, range, robust mean absolute deviation, root mean squared, skewness, total energy, uniformity, variance, and standard deviation were extracted from the ADC, T1, T2, and PD maps using the open-source Pyradiomics package (https://pypi.org/project/pyradiomics/) [22].

### Inter- and intra-observer reliability analysis

To assess inter-observer reliability, radiologists 1 and 2 independently delineated VOIs for 100 randomly selected patients in the training cohort. Radiologist 1 repeated the delineation of the same 100 patients after 1 month to assess intra-observer reliability.

### Treatment procedure and tumor response evaluation to IC

Baseline MRI was performed approximately 1.0 day (range 0–3 days) before IC initiation. The specific chemotherapy regimen is detailed in the Supplementary Materials.

Radiologist 5, a senior radiologist with 23 years of experience in neck imaging diagnosis, blinded to clinicopathological data, assessed IC response for all patients and participants using before and after IC nasopharyngeal MRI. Treatment responses were categorized per RECIST 1.1 as: *complete response* in the case of lesion disappearance with pathological lymph node short-axis < 10 mm [21]; *partial response* in the case of ≥ 30% decrease in target lesion diameters; *progressive disease* in the case of ≥ 20% increase in target lesion diameters or the appearance of new lesions; and *stable disease* for as any other outcome. Responders included patients with complete or partial response, while non-responders were those with stable or progressive disease.

### Development of predictive models and statistical analysis

Variables with a *p* value < 0.05 in univariable analysis were included in a stepwise backward multivariable analysis. Based on this analysis, variables with a *p* value < 0.05 were used to develop models for predicting IC efficacy. Using logistic regression, a clinicopathological model, an imaging model based on significant histogram features from ADC, T1, T2, and PD maps, and a nomogram, integrating the most significant predictors, were developed. All three models were independently evaluated in the test cohort for IC response.

The performance of each model in predicting IC response was assessed using receiver operating characteristic curve analysis, with area under the curve (AUC) values computed. The DeLong method was used to compare the AUC values between different models.

Additionally, an interactive online nomogram was developed to enable clinicians to easily assess the likelihood of IC response by entering relevant features. (https://huangyao96.shinyapps.io/DynNomapp-Magic/).

Statistical analyses were performed using Python (version 3.9.5) and R (version 4.3.1). Continuous variables were presented as medians and interquartile ranges, and categorical variables as frequencies and percentages. The normality of the continuous variables was evaluated using the Kolmogorov–Smirnov test. For group comparisons, the Student’s *t*-test or the Wilcoxon *U*-test was used for continuous variables, and χ^2^ or Fisher's exact tests were employed for categorical variables. Two-sided *p* values < 0.05 were considered statistically significant. Intraclass correlation coefficients were used to assess the histogram parameter agreement.

A minimum sample size of 59 patients was determined based on the following parameters: a statistical power of 90%, a two-sided significance level of 0.05, an alternative hypothesis AUC of 0.75, and a null hypothesis AUC of 0.50.

## Results

### Patients characteristics

A total of 267 NPC patients were enrolled in this study [male: 187 (70.0%); mean age: 52.2 (range: 43.5–58.7) years], with their characteristics outlined in Table S2. As illustrated in Fig. 1, the training set comprised 185 patients: 53 non-responders (50 SD, 3 progressive disease) and 132 responders (101 partial response, 31 complete response); the test set comprised 82 patients: 27 non-responders (27 stable disease, 0 progressive disease) and 55 responders (43 partial response, 12 complete response). Univariate analyses of the training cohort identified TSR as the only clinicopathological feature associated with treatment response that showed a significant difference between the response and non-response groups (*p* = 0.013), as shown in Table 1.

**Table 1:** Characteristics of participants in the training cohort

Characteristics	Total (*n* = 185)	Responders (*n* = 132)	Non-responders (*n* = 53)	*p* value
Age (years)	52.0 (42.9–59.4)	51.5 (43.0–58.3)	52.3 (39.8–62.4)	0.966^#^
Sex (*n*, %)				0.723^*^
Male	136 (73.5)	98 (74.2)	38 (71.7)	
Female	49 (26.5)	34 (25.8)	15 (28.3)	
NLR	2.60 (2.15–3.59)	2.59 (1.96–3.61)	2.75 (2.00–3.52)	0.995^#^
PLR	152.59 (115.03–196.87)	157.66 (115.28–198.23)	142.73 (116.33–196.01)	0.544^#^
SII	599.12 (422.16–923.40)	599.31 (430.33–941.47)	590.03 (417.57–862.99)	0.932^#^
T stage (*n*, %)				0.671^*^
T1	13 (7.0)	11 (8.3)	2 (3.8)	
T2	29 (15.7)	20 (15.2)	9 (17.0)	
T3	87 (47.0)	61 (46.2)	26 (49.1)	
T4	56 (30.3)	40 (30.3)	16 (30.1)	
N stage (*n*, %)				0.728^*^
N0	7 (3.8)	6 (4.5)	1 (1.9)	
N1	81 (43.8)	56 (42.4)	25 (47.2)	
N2	59 (31.9)	43 (32.6)	16 (30.2)	
N3	38 (20.5)	27 (20.5)	11 (20.7)	
AJCC stage (*n*, %)				0.244^*^
III	97 (52.4)	58 (43.9)	27 (50.9)	
IVa	88 (47.6)	74 (56.1)	26 (49.1)	
EBV DNA (*n*, %)				0.069^*^
< 4,000	85 (45.9)	54 (40.9)	31 (58.5)	
≥ 4,000	100 (54.1)	78 (59.1)	22 (41.5)	
TSR (≥ 50%) (*n*, %)	127 (68.6)	97 (73.5)	30 (56.6)	0.013^*^

Patient characteristics were compared using the Mann–Whitney *U*-test (#), Fisher's exact test or χ^2^ test (*) as appropriate. T and N stages were determined according to the 8th edition American Joint Committee on Cancer staging system for head and neck cancer

*EBV DNA* Copy number of Epstein–Barr virus DNA, *NLR* Neutrophil-to-lymphocyte ratio, *PLR* Platelet-to-lymphocyte ratio, *SII* Systemic immune inflammation index, *TSR* Tumor-stroma ratio

Inter- and intra-observer agreement for histogram parameters derived from primary tumors showed good to excellent reliability, with intraclass correlation coefficients ranging from 0.774 (95% confidence interval [CI]: 0.709–0.859) to 0.923 (95% CI: 0.855–0.945), respectively.

### Predictors of IC response

TSR (OR 2.131, 95% CI: 1.070–4.242; *p* = 0.031), T1map_mean (OR 0.688, 95% CI: 0.483–0.980; *p* = 0.038), and PDmap_Kurtosis (OR 0.643, 95% CI: 0.448–0.923; *p* = 0.017) emerged as independent predictors of IC response (Table 2). This study did not find any histogram features derived from pretreatment T2 map and ADC associated with IC response (Table S3). The differences of ADC_mean, T1map_mean, T2map_mean, PDmap_mean, and PDmap_Kurtosis between the response and non-response groups to IC are presented in Supplementary Fig. S1.

**Table 2:** Multivariable analysis of clinicopathological and imaging characteristics associated with IC response in the training cohort

Characteristics	Multivariable	*p*-value
	Odds ratio (95% CI)	
TSR ≥ 50% (< 50% as reference)	2.131 (1.070, 4.242)	**0.031**
T1map_Mean	0.688 (0.483, 0.980)	**0.038**
T2map_Maximum	0.722 (0.544, 1.494)	0.231
T2map_TotalEnergy	1.132 (0.560, 1.447)	0.342
T2map_Variance	0.866 (0.310, 1.079)	0.326
T2map_StandardDeviation	4.332 (0.793, 4.972)	0.220
PDmap_Kurtosis	0.643 (0.448, 0.923)	**0.017**

Bolded values indicate *p* < 0.05

*PD* Proton density, *TSR* Tumor-stroma ratio

### Performance of single-parameter models in discriminating responders from non-responders

**Fig. 2:**
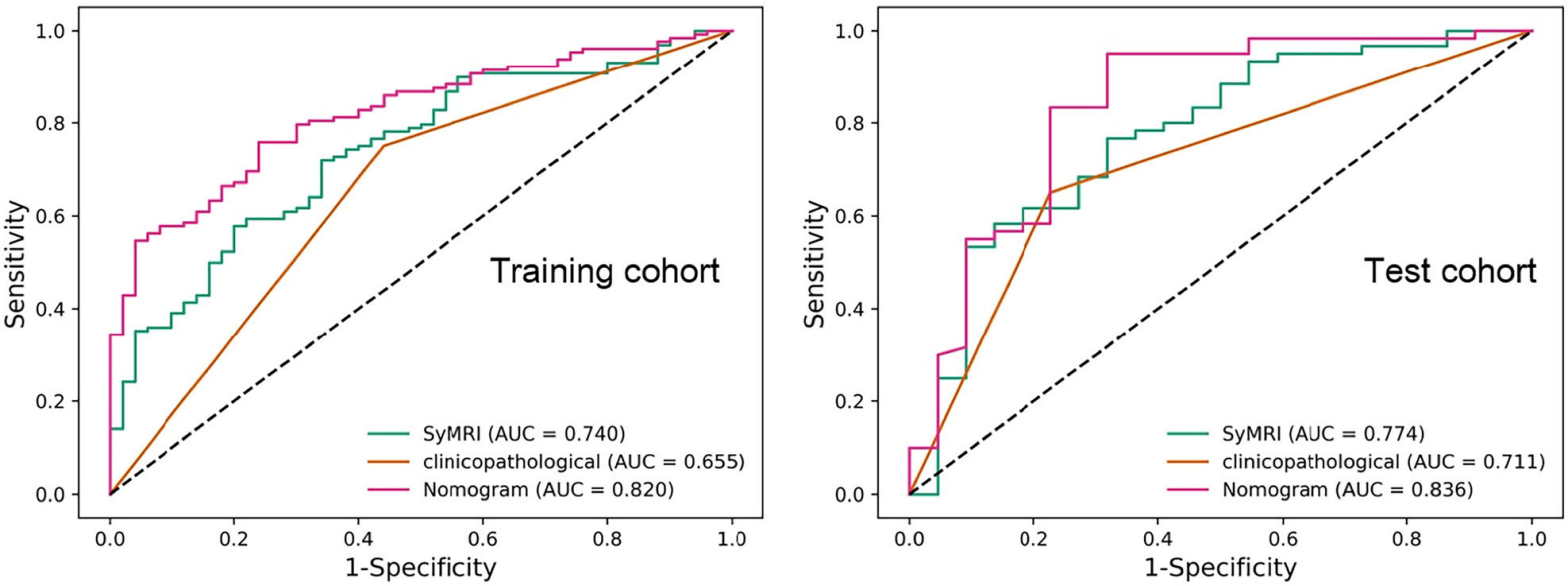
Receiver operating characteristic curves illustrating the performance of the clinicopathological, TSR, synthetic SyMRI, and nomogram models in predicting IC response in both the training and test cohorts. AUC, Area under the curve

In classifying IC responders, the clinicopathological model based on TSR showed an AUC of 0.655 (95% CI: 0.589–0.782) in the training cohort and 0.711 (95% CI: 0.577–0.809) in the test cohort (Table 3 and Fig. 2). The SyMRI model using T1map_mean and PDmap_Kurtosis demonstrated an AUC of 0.740 (95% CI: 0.613–0.831) in the training cohort and 0.774 (95% CI: 0.623–0.822) in the test cohort (Table 3 and Fig. 2).

**Table 3:** Performance of the TSR, SyMRI, and nomogram models in predicting IC response in the training and test cohorts

Cohorts	Models	AUC	Sensitivity	Specificity
Training cohort	TSR	0.655 (0.589, 0.782)	0.733 (0.667, 0.791)	0.712 (0.643, 0.753)
	SyMRI	0.740 (0.644, 0.769)	0.658 (0.613, 0.815)	0.811 (0.701, 0.876)
	Nomogram	0.820 (0.751, 0.866)	0.798 (0.671, 0.844)	0.826 (0.677, 0.902)
Test cohort	TSR	0.711 (0.577, 0.809)	0.693 (0.634, 0.769)	0.778 (0.684, 0.880)
	SyMRI	0.774 (0.623, 0.822)	0.604 (0.574, 0.655)	0.894 (0.759, 0.940)
	Nomogram	0.836 (0.690, 0.914)	0.795 (0.723, 0.879)	0.852 (0.784, 0.913)

*AUC* Area under the curve, *SyMRI* Synthetic magnetic resonance imaging, *TSR* Tumor-stroma ratio.

### Performance of the nomogram for predicting response to IC

The nomogram, which integrated TSR, T1map_mean, and PDmap_Kurtosis demonstrated superior predictive performance, achieving an AUC of 0.820 (95% CI: 0.751–0.866) in the training cohort and 0.836 (95% CI: 0.690–0.914) in the test cohort (Table 3 and Fig. 2).

TheDeLong test confirmed that the combined model significantly outperformed the clinicopathological model (TSR) and SyMRI model alone in predicting IC response, with statistically significant improvements in AUC observed in the training (*p* = 0.021, *p* = 0.038) and test cohorts (*p* = 0.015, *p* = 0.003) (Supplementary Table S4).

**Fig. 3:**
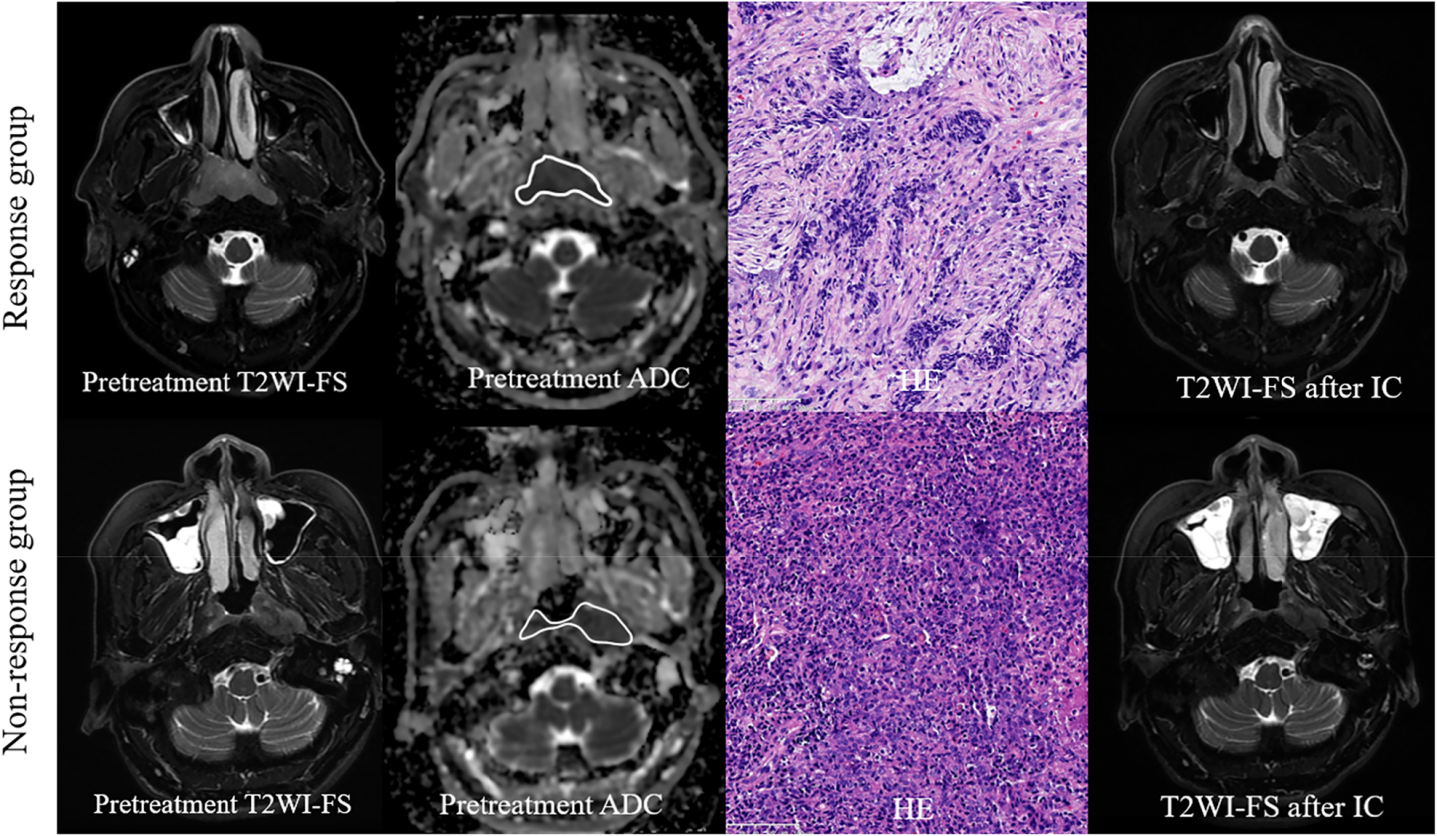
Representative images of two patients with NPC. First row: a 55-year-old male patient with NPC (T3N2M0, 8th AJCC stage III) was classified as having a partial response (response group) according to RECIST 1.1 criteria after three cycles of IC. The chemotherapy regimen consisted of liposomal paclitaxel 260 mg on day 1, cisplatin 50 mg on days 1–2, and capecitabine 1.5 g in the morning and 2.0 g in the evening from days 1–14. HE-stained slide (20×) revealed a TSR greater than 50%. Second row: a 49-year-old male patient with NPC (T3N2M0, 8th AJCC stage III) was classified as having stable disease according to RECIST 1.1 criteria after three cycles of IC. The chemotherapy regimen consisted of liposomal paclitaxel 260 mg on day 1 and oxaliplatin 50 mg on day 1. HE-stained slide (20×) showed a TSR less than 50%. Note the regions of interest from a single slice on the ADC maps. ADC, Apparent diffusion coefficient; AJCC, American Joint Committee on Cancer; HE, Hematoxylin and eosin; IC, Induction chemotherapy; NPC, Nasopharyngeal carcinoma; RECIST, Response Evaluation Criteria in Solid Tumors; T2WI-FS, Fat-saturated T2-weighted imaging

Figure 3 presents representative images showing the VOI delineation and hematoxylin and eosin-stained slides for responders and non-responders.

**Fig. 4:**
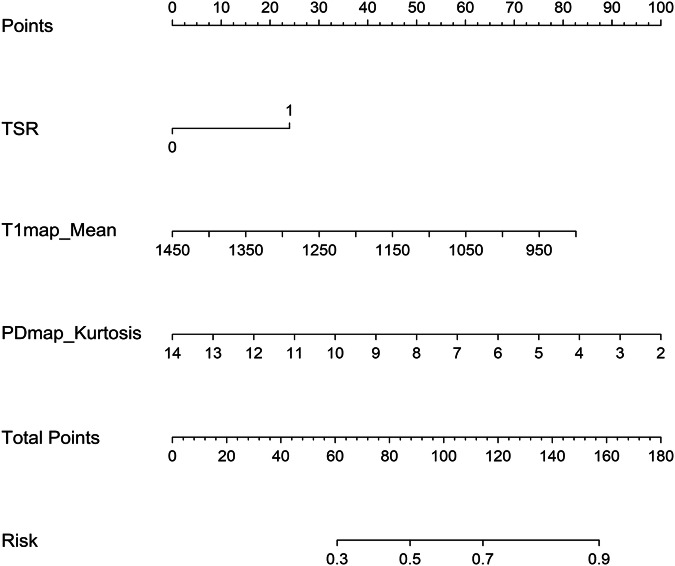
Nomogram model for predicting IC response. The nomogram model formula was as follows: “Logit “(“P”)” = 7.782﹣0.005·T1map_mean﹣0.260·PDmap_Kurtosis + 0.674·TSR”. The coefficients of the model formula were estimated using the maximum likelihood estimation method

The optimal cutoff value for the nomogram score was 131.418. Patients with a low

pretreatment T1map_mean, PDmap_Kurtosis, and a high TSR were more likely to be classified into the response group after IC (Fig. 4).

## Discussion

In this study, we evaluated the predictive potential of TSR and histogram parameters from pretreatment SyMRI and ADC in assessing IC response in patients with locoregionally advanced NPC. Our findings indicated that histogram parameters from pretreatment ADC and T2map were not reliable predictors of IC response. However, the nomogram incorporating TSR along with pretreatment SyMRI-derived T1map_mean and PDmap_Kurtosis showed high predictive performance in the training and test cohorts, with AUC of 0.820 and 0.836, respectively. The model developed in this study could effectively identify patients who are suitable for IC.

TSR has emerged as a significant histologic marker for predicting therapeutic response and prognosis across various solid tumors. Breast cancer studies have shown that patients with high TSR (low stroma content) exhibit high pathologic response rates to neoadjuvant chemotherapy [23], and the results in ovarian cancer were similarly observed [24]. Our study aligns with these findings, suggesting that high stroma content within tumors may be associated with abundant cancer-associated fibroblasts, which have been implicated in promoting chemoresistance [25]. Cancer‑associated fibroblast-derived exosomes can facilitate drug efflux or sequestration in tumor cells, thereby reducing chemotherapy efficacy.

Quantitative T1 values are influenced by various factors associated with tissue composition, such as macromolecule concentration, hydration state, and tissue water content. In this study, the low T1map_mean of the primary tumors was associated with the IC response. This finding aligns with previously reported outcomes in breast cancer following neoadjuvant chemotherapy [26]. The finding may be attributed to diminished cellular proliferation [27] and rich stroma [28] within tumors exhibiting lower T1 relaxation times, potentially leading to reduced cellular density and consequent chemosensitivity. Prior research has demonstrated no significant association between PD value and neoadjuvant chemotherapy response in breast cancer [26], a finding corroborated by our study in NPC. Kurtosis, a measure of the ‘peakedness’ of distribution within a VOI, provides insight into intratumoral heterogeneity. Our result revealed a significantly lower PDmap_kurtosis value in the response group than in the non-response group, suggesting that decreased intratumoral heterogeneity may improve treatment response. This was consistent with prior results on intratumoral heterogeneity in NPC [29, 30] and breast cancer [31].

Although the role of ADC in predicting treatment outcomes in various tumors has been widely studied [32, 33], its predictive value for chemotherapy efficacy in NPC remains inconsistent. Tu et al [34] associated a higher pretreatment ADC_mean with increased chemotherapy sensitivity; however, consistent with prior findings, our study demonstrated that pretreatment ADC_mean did not predict IC response [9], presumably due to limitations in ADC measurements derived from DWI, which often assumes Gaussian diffusion, conflating the effects of various factors, such as tissue cell count, fluid viscosity, membrane permeability, and blood flow [17].

Previous studies have used radiomic features derived from conventional sequences (T2-weighted and contrast-enhanced T1-weighted images) to predict the efficacy of IC [35, 36]. In contrast, our study employed histogram features because they are relatively easier to implement than radiomics and offer greater interpretability for clinicians. In addition, our nomogram outperformed both the clinicopathological model and SyMRI model, demonstrating its ability to effectively integrate information across different dimensions. Furthermore, we developed an open-source nomogram and provided a web application for clinical use and validation.

The study had several limitations. First, as SyMRI is a relatively new technique and many institutions do not yet have such data, the single-center nature of this study may limit the generalizability of the results; therefore, multicenter studies are needed to validate the stability and reproducibility of the findings. The relatively small sample size of the test cohort limits the robustness of our findings, necessitating larger cohorts in future studies. Second, inherent variations in tissue biopsy sampling may introduce inconsistencies in TSR assessments, as tissue biopsies cannot fully represent the entire tumor, potentially reducing the model’s predictive accuracy. Third, the DWI MUSE sequence was acquired with a relatively small matrix size (96 × 96), which might have affected image quality. Fourth, RECIST focuses solely on tumor size for assessing treatment response and overlooks functional changes after chemotherapy. As is well known, a reliable and objective method has yet to be established; therefore, RECIST remained the reference standard for assessing treatment response [37].

In conclusion, our study highlighted that baseline SyMRI metrics (including T1map_mean and PDmap_Kurtosis) and TSR were reliable biomarkers for predicting IC response in locally advanced NPC. Based on these three variables, we developed a nomogram that exhibited robust performance in predicting IC response.

## Supplementary information

**Additional file 1:** **Fig. S1** Comparison of five pretreatment synthetic magnetic resonance imaging histogram features between response and non-response groups after IC in NPC patients. *ADC* Apparent diffusion coefficient, *NPC* Nasopharyngeal carcinoma, *PD* Proton density. Difference testing of ADC_mean, T2map_mean, PDmap_mean, and PDmap_Kurtosis was performed using the Mann–Whitney *U*-test, and T1map_mean using the *t*-test. **Table S1** MRI protocol. **Table S2** Characteristics of participants in the training and the test cohorts. **Table S3** Univariable analysis of the associations of clinicopathological and imaging characteristics with IC response in the training cohort. **Table S4:** Comparison of the performance of the TSR, SyMRI, and nomogram models in predicting IC response in the training and test cohorts.

## Data Availability

The datasets generated and/or analyzed during the current study are not publicly available because the subjects did not provide written consent for their data to be publicly shared. However, datasets can be obtained by request from specific research groups.

## Abbreviations

ADC: Apparent diffusion coefficient
AUC: Area under curve
CI: Confidence interval
IC: Induction chemotherapy
MRI: Magnetic resonance imaging
NPC: Nasopharyngeal carcinoma
PD: Proton density
RECIST: Response evaluation criteria in solid tumors
SyMRI: Synthetic MRI
TSR: Tumor-stroma ratio
VOI: Volume of interest
