# Supplementary material for: Predicting induction chemotherapy response based on tumor-stroma ratio and pretreatment synthetic MRI in nasopharyngeal carcinoma
Source: Eur Radiol Exp. 2026 Feb 24;10:20. doi: 10.1186/s41747-026-00683-5 (PMC12932746; doi:10.1186/s41747-026-00683-5)
Supplement: Supplementary file 1 — Additional file 1: Fig. S1 Comparison of five pretreatment synthetic magnetic resonance imaging histogram features between response and non-response groups after IC in NPC patients. ADC Apparent diffusion coefficient, NPC Nasopharyngeal carcinoma, PD Proton density. Difference testing of ADC_mean, T2map_mean, PDmap_mean, and PDmap_Kurtosis was performed using the Mann–Whitney U-test, and T1map_mean using the t-test. Table S1 MRI protocol. Table S2 Characteristics of participants in the training and the test cohorts. Table S3 Univariable analysis of the associations of clinicopathological and imaging characteristics with IC response in the training cohort. Table S4: Comparison of the performance of the TSR, SyMRI, and nomogram models in predicting IC response in the training and test cohorts. [file 41747_2026_683_MOESM1_ESM.pdf]

Predicting Induction Chemotherapy Response Based on Tumor-stroma Ratio and Pretreatment Synthetic MRI in Nasopharyngeal Carcinoma

ELECTRONIC SUPPLEMENTARY MATERIAL

TSR assessment

Following the guidelines we first scanned the whole tumor at low magnification (×5 objective) to select an area with the highest amount of tumor-associated stroma. Then, at higher magnification using a ×10 objective we assessed an area of 3 mm^2 for the amount of tumor associated stroma so that cancer cells were present in all 4 edges of the selected microscopic field. Only a stromal area surrounded by cancer cells was assessed to ensure that tumor-associated stroma only was evaluated. In cases of a heterogenous tumor with areas of both high and low amounts of tumor-associated stroma, the stroma-high area was considered decisive for scoring the case, as recommended in the guidelines [20].

EBV DNA

Patients were subsequently stratified into EBV DNA high (≥ 4000 copy number) and low (< 4000 copy number) groups, based on prior literature suggesting that different levels of EBV DNA are associated with varying sensitivities to induction chemotherapy [36].

Induction chemotherapy regimens

The induction chemotherapy regimen included two or three cycles (35–73 days) of GP (gemcitabine 1000 mg/m^2 on days 1 and 8; cisplatin 80 mg/m^2 on day 1), TPC (paclitaxel 135–175 mg/m^2 on day 1; cisplatin 80 mg/m^2 on day 1; capecitabine 1,000 mg/m^2 twice daily on days 1–14), TP (paclitaxel 135–175 mg/m^2 on day 1; cisplatin 80 mg/m^2 on day 1), DPF (docetaxel 75 mg/m^2 and cisplatin 80 mg/m^2 on day 1; fluorouracil 750 mg/m^2 on days 1–5), or DP (docetaxel 75 mg/m^2 and cisplatin 80 mg/m^2 on day 1).

Table S1 MRI protocol

Parameters	Axial T1WI	Axial T2WI	Axial T2WI-FS	MUSE DWI	Axial SyMRI
Sequence	FSE	FSE	FSE	EPI	MAGiC
TR (ms)	600	5300	5300	2400	4000
TE (ms)	Min ful	85	85	Min	19.3
FOV (cm)	22	22	22	22	24
Acquisition matrix	320*320	400*400	320*240	96*96	256*256
Slice thickness/gap (mm)	4/0.4	3/0.3	3/0.3	5/0.5	3/0.3
Flip angle	111	111	111	90	90
bandwidth	83.33	62.5	62.5	250	20.83
NEX	3	3	2	2	1
Acquisition time	1:40	2:04	2:19	1:22	4:32
b values (sec/mm^2)	N/A	N/A	N/A	0/800	N/A

DWI Diffusion-weighted imaging, *EPI* Echo-planar imaging, *FOV* Field of view, *FS* Fat saturation, *FSE* Fast spin-echo, *MRI* Magnetic resonance imaging, *NEX* Number of excitations, *SyMRI* Synthetic MRI, *T1WI* T1-weighted imaging, *T2WI* T2-weighted imaging, *TE* Echo time, *TR* Repetition time.

Table S2 Characteristics of participants in the training and the test cohorts

Characteristics	Total (*n*=267)	Training cohort (*n* = 185)	Test cohort (*n* = 82)	*P* value
**Age** (years)	52.2 (43.5-58.7)	52.0 (42.9-59.4)	53.4 (45.3-58.5)	0.240#
**Sex** (n, %)				0.623*
Male	187 (70.0)	136 (73.5)	55 (67.1)	
Female	80 (30.0)	49 (26.5)	27 (32.9)	
**NLR**	2.58 (1.92-3.53)	2.60 (2.15-3.59)	2.47 (1.89-3.09)	0.427#
**PLR**	154.55 (115.28-202.15)	152.59 (115.03-196.87)	156.92 (116.24-208.39)	0.570#
**SII**	586.89 (422.91-884.34)	599.12 (422.16-923.40)	561.17 (450.90-832.38)	0.778#
**T stage** (n, %)				0.910*
T1	20 (7.5)	13 (7.0)	7 (8.5)	
T2	49 (18.3)	29 (15.7)	20 (24.4)	
T3	118 (44.2)	87 (47.0)	31 (37.8)	
T4	80 (30.0)	56 (30.3)	24 (29.3)	
**N stage** (n, %)				0.461*
N0	11 (4.1)	7 (3.8)	6 (7.3)	
N1	112 (41.9)	81 (43.8)	34 (41.5)	
N2	86 (32.3)	59 (31.9)	23 (28.0)	
N3	58 (21.7)	38 (20.5)	19 (23.2)	
**AJCC stage** (n, %)				0.401*
III	136 (50.9)	97 (52.4)	39 (47.6)	
IVa	131 (49.1)	88 (47.6)	43 (52.4)	
**EBV DNA** (n, %)				0.745*
<4000	118 (44.2)	85 (45.9)	33 (40.2)	
≥4000	149 (55.8)	100 (54.1)	49 (59.8)	
**TSR (≥50%)** (n, %)	174 (65.2)	127 (68.6)	47 (57.3)	0.317*
**Responders** (n, %)	181 (67.8)	132 (71.4)	55 (67.1)	0.605*

Data are medians, with interquartile range in parentheses. Patient characteristics were compared using the Mann-Whitney *U* test (#), Fisher exact test or χ^2 test (*) as appropriate. T and N stage were determined according to the eighth edition m a single slice on the ADC maps. staging system for head and neck cancer. *EBV DNA* Copy number of Epstein–Barr virus DNA, *NLR* Neutrophil-to-lymphocyte ratio, *PLR* Platelet-to-lymphocyte ratio, *SII* Systemic immune inflammation index, *TSR* tumor stroma ratio.

Table S3 Univariable analysis of the associations of clinicopathological and imaging characteristics with induction chemotherapy response in the training cohort

Characteristics	Univariable analysis	
	Odds ratio (95% CI)	*p*-value
Clinicopathological characteristics		
Age	0.999 (0.971,1.028)	0.956
Sex		
Male	Reference	
Female	0.843 (0.410,1.735)	0.644
T stage		
T1	Reference	
T2	1.137(0.4449,2.885)	0.786
T3	0.741(0.384,1.427)	0.370
T4	1.061(0.521,2.60)	0.871
N stage		
N0	Reference	
N1	0.753(0.391,1.451)	0.397
N2	1.038(0.516,2.089)	0.917
N3	1.274(0.532,3.052)	0.586
Clinical stage		
Ⅲ	Reference	
Ⅳa	0.784(0.524,1.251)	0.349
EBV DNA		
<4000	Reference	
≥4000	1.035(0.513,2.085)	0.923
TSR		
<50%	Reference	
≥50%	2.165(1.114,4.206)	0.022
Imaging characteristics		
T1map_10Percentile	1.099 (0.723, 1.331)	0.533
T1map_90Percentile	0.679 (0.355, 1.401)	0.720
T1map_Energy	0.892 (0.712, 1.244)	0.704
T1map_Entropy	1.002 (0.667, 1.383)	0.819
T1map_InterquartileRange	0.655 (0.367,1.631)	0.529
T1map_Kurtosis	0.207 (0.029, 1.458)	0.308
T1map_Maximum	0.832 (0.535, 1.294)	0.294
T1map_MeanAbsoluteDeviation	1.048 (0.796, 1.380)	0.743
T1map_Mean	0.884 (0.690, 0.996)	0.026
T1map_Median	0.748 (0.393, 1.427)	0.956
T1map_Minimum	0.837 (0.469, 1.492)	0.252
T1map_Range	1.005 (0.998, 1.012)	0.602
T1map_RobustMeanAbsoluteDeviation	1.523 (0.065, 35.875)	0.707
T1map_RootMeanSquared	1.111 (0.915, 1.350)	0.704

T1map_Skewness	2.275 (0.984, 4.575)	0.884
T1map_TotalEnergy	0.035 (0.003, 1.021)	0.980
T1map_Uniformity	4.145 (0.204, 84.251)	0.930
T1map_Variance	0.106 (0.007, 1.636)	0.876
T1map_StandardDeviation	0.768 (0.617, 1.216)	0.449
T2map_10Percentile	2.824 (0.783, 6.078)	0.774
T2map_90Percentile	0.631 (0.429, 1.172)	0.160
T2map_Energy	0.158 (0.026, 1.439)	0.360
T2map_Entropy	1.515 (0.743, 1.376)	0.722
T2map_InterquartileRange	3.182 (0.434, 8.281)	0.102
T2map_Kurtosis	0.686 (0.482, 1.499)	0.787
T2map_Maximum	0.791 (0.639, 0.981)	0.036
T2map_MeanAbsoluteDeviation	1.956 (0.814, 4.202)	0.162
T2map_Mean	2.477 (0.803, 5.028)	0.332
T2map_Median	3.182 (0.434, 8.281)	0.103
T2map_Minimum	0.317 (0.122, 1.634)	0.121
T2map_Range	0.562 (0.388, 2.284)	0.376
T2map_RobustMeanAbsoluteDeviation	0.613 (0.155, 1.252)	0.579
T2map_RootMeanSquared	1.259 (0.102, 3.718)	0.084
T2map_Skewness	2.354 (0.842, 6.126)	0.167
T2map_TotalEnergy	1.695 (1.142, 1.802)	0.047
T2map_Uniformity	0.779 (0.495, 1.741)	0.355
T2map_Variance	0.978 (0.964, 0.991)	0.034
T2map_StandardDeviation	5.144 (1.864, 8.196)	0.030
PDmap_10Percentile	1.394 (0.502, 2.771)	0.766
PDmap_90Percentile	1.178 (0.348, 3.221)	0.713
PDmap_Energy	2.014 (0.493, 3.278)	0.117
PDmap_Entropy	0.247 (0.009, 1.748)	0.722
PDmap_InterquartileRange	0.543 (0.176, 1.537)	0.171
PDmap_Kurtosis	0.756 (0.615, 0.929)	0.008
PDmap_Maximum	1.812 (0.399, 1.743)	0.894
PDmap_MeanAbsoluteDeviation	0.435 (0.142, 1.816)	0.804
PDmap_Mean	0.933 (0.107, 2.401)	0.671
PDmap_Median	0.325 (0.101, 1.273)	0.296
PDmap_Minimum	3.279 (0.553, 6.879)	0.344
PDmap_Range	0.993 (0.476, 2.517)	0.336
PDmap_RobustMeanAbsoluteDeviation	0.077 (0.002, 1.023)	0.767
PDmap_RootMeanSquared	0.872 (0.642, 1.454)	0.832
PDmap_Skewness	0.710 (0.259, 1.092)	0.167
PDmap_TotalEnergy	0.257 (0.162, 2.457)	0.173
PDmap_Uniformity	1.833 (0.564, 3.044)	0.755
PDmap_Variance	1.464 (0.733, 4.732)	0.453

PDmap_StandardDeviation	0.280 (0.045, 1.473)	0.126
ADC_10Percentile	1.099 (0.723, 1.331)	0.533
ADC_90Percentile	0.679 (0.355, 1.401)	0.720
ADC_Energy	0.892 (0.712, 1.244)	0.704
ADC_Entropy	1.002 (0.667, 1.383)	0.819
ADC_InterquartileRange	0.655 (0.367,1.631)	0.529
ADC_Kurtosis	0.207 (0.029, 1.458)	0.308
ADC_Maximum	0.832 (0.535, 1.294)	0.294
ADC_MeanAbsoluteDeviation	1.048 (0.796, 1.380)	0.743
ADC_Mean	2.434 (0.795, 2.858)	0.403
ADC_Median	0.748 (0.393, 1.427)	0.956
ADC_Minimum	0.837 (0.469, 1.492)	0.252
ADC_Range	1.005 (0.998, 1.012)	0.602
ADC_RobustMeanAbsoluteDeviation	1.523 (0.065, 3.875)	0.707
ADC_RootMeanSquared	1.111 (0.915, 1.350)	0.704
ADC_Skewness	2.275 (0.984, 4.575)	0.884
ADC_TotalEnergy	0.035 (0.003, 1.021)	0.980
ADC_Uniformity	4.145 (0.204, 8.251)	0.930
ADC_Variance	0.106 (0.007, 1.636)	0.876
ADC_StandardDeviation	0.280 (0.045, 1.473)	0.126

ADC Apparent diffusion coefficient; *CI* Confidence interval, *EBV DNA* Copy number of Epstein–Barr virus DNA, *PD* Proton density, *TSR* Tumor-stroma ratio. Bolded values indicate $p < 0.05$.

Table S4: Comparison of the performance of the TSR, SyMRI, and nomogram models in predicting induction chemotherapy response in the training and test cohorts

Cohorts	Models	TSR	SyMRI	nomogram
Training cohort	TSR	–	0.039	0.021
	SyMRI	0.039	–	0.038
	Nomogram	0.021	0.038	–
Test cohort	TSR	–	0.044	0.015
	SyMRI	0.044	–	0.003
	Nomogram	0.015	0.003	–

SyMRI Synthetic magnetic resonance imaging, *TSR* Tumor-stroma ratio. The numbers in the table represent the *p*-values obtained using the Delong test for two models.

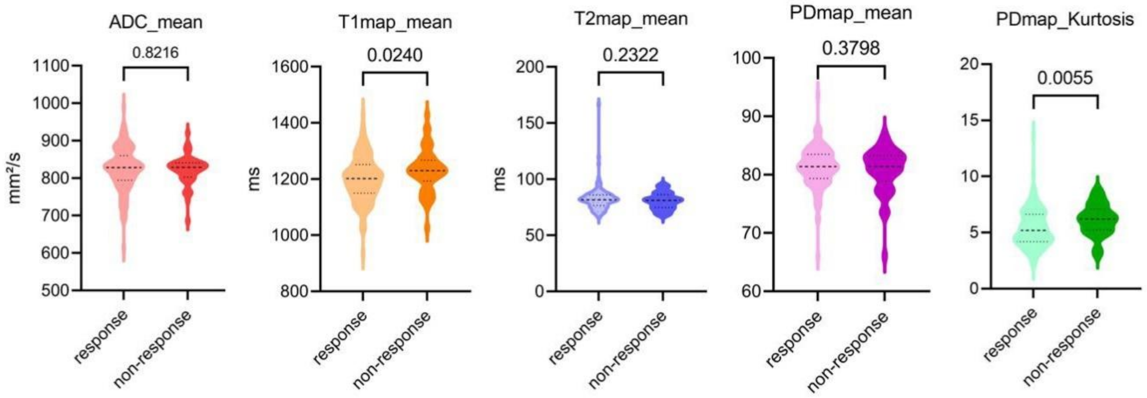

Fig. S1 Comparison of five pretreatment synthetic magnetic resonance imaging histogram features between response and non-response groups after induction chemotherapy in NPC patients. *ADC* Apparent diffusion coefficient, *NPC* Nasopharyngeal carcinoma, *PD* Proton density. Difference testing of ADC_mean, T2map_mean, PDmap_mean, and PDmap_Kurtosis was performed using the Mann-Whitney *U* test, T1map_mean using the *t* test.

References

20. Almangush A, Ruuskanen M, Hagström J, et al. Prognostic Significance of Tumor-associated Stroma in Nasopharyngeal Carcinoma: A Multicenter Study. Am J Surg Pathol 2024;48:54-58.
36. Li XY, Luo DH, Guo L et al. Deintensified Chemoradiotherapy for Pretreatment Epstein-Barr Virus DNA-Selected Low-Risk Locoregionally Advanced Nasopharyngeal Carcinoma: A Phase II Randomized Noninferiority Trial. J Clin Oncol 2022;40:1163–1173.
